# MdPP2C24/37, Protein Phosphatase Type 2Cs from Apple, Interact with MdPYL2/12 to Negatively Regulate ABA Signaling in Transgenic *Arabidopsis*

**DOI:** 10.3390/ijms232214375

**Published:** 2022-11-19

**Authors:** Ying-Ying Liu, Wen-Sen Shi, Yu Liu, Xue-Meng Gao, Bo Hu, Hao-Ran Sun, Xiao-Yi Li, Yi Yang, Xu-Feng Li, Zhi-Bin Liu, Jian-Mei Wang

**Affiliations:** Key Laboratory of Bio-Resources and Eco-Environment of Ministry of Education, College of Life Sciences, Sichuan University, Chengdu 610065, China

**Keywords:** *Malus domestica*, abscisic acid, drought stress, MdPP2C, MdPYL

## Abstract

The phytohormone abscisic acid (ABA) plays an important role in the ability of plants to cope with drought stress. As core members of the ABA signaling pathway, protein phosphatase type 2Cs (PP2Cs) have been reported in many species. However, the functions of MdPP2Cs in apple (*Malus domestica*) are unclear. In this study, we identified two PP2C-encoding genes, *MdPP2C24/37*, with conserved PP2C catalytic domains, using sequence alignment. The nucleus-located *MdPP2C24/37* genes were induced by ABA or mannitol in apple. Genetic analysis revealed that overexpression of *MdPP2C24/37* in *Arabidopsis thaliana* led to plant insensitivity to ABA or mannitol treatment, in terms of inhibiting seed germination and overall seedling establishment. The expression of stress marker genes was upregulated in *MdPP2C24/37* transgenic lines. At the same time, *MdPP2C24/37* transgenic lines displayed inhibited ABA-mediated stomatal closure, which led to higher water loss rates. Moreover, when exposed to drought stress, chlorophyll levels decreased and MDA and H_2_O_2_ levels accumulated in the *MdPP2C24/37* transgenic lines. Further, MdPP2C24/37 interacted with MdPYL2/12 in vitro and vivo. The results indicate that *MdPP2C24/37* act as negative regulators in response to ABA-mediated drought resistance.

## 1. Introduction

The apple tree (*Malus domestica*) is one of the most important fruit trees in the world, and its yield is greatly affected by adverse environmental cues, such as drought stress [1,2]. As the world’s largest apple producer, China accounts for 50% of the world’s apple production, among which ‘*huashuo*’ is an elite hybrid cultivar [3]. Whole genome sequencing analysis has revealed accurate apple gene sequences and provides an effective method to study the function of stress-responsive genes [4,5]. Thus, the study of the apple’s resistance to drought stress is urgently needed to cultivate various apple trees with excellent traits, such as ‘*huashuo*’. 

Unlike animals that can escape adverse environments, sessile plants have developed a series of defensive mechanisms to deal with various abiotic stresses, such as drought, cold, and heat [6]. As a major environmental factor, drought stress affects the geographical distribution of agricultural productivity and environmental sustainability, causing abscisic acid (ABA) accumulation in plants. Further, the phytohormone ABA regulates many physiological processes, including plant development, seed maturation, stomata movement, and signal transduction under abiotic stress [7,8].

Many key components in the ABA signaling pathway have been identified at the molecular level, including ABA receptors, group A type 2C protein phosphatases (PP2Cs), SnRKs (sucrose non-fermenting-1 related protein kinases), transcription factors, and ion channels [9,10,11]. When ABA receptors PYR1/PYL/RCAR (pyrabactin resistance 1/PYR1-like/regulatory components of ABA receptor) perceive ABA, they bind directly to PP2Cs. This reaction inhibits the activity of PP2Cs. Later, the inhibited PP2Cs release the bound SnRKs, causing SnRKs to shuttle from inactivated status to activated status in order to phosphorylate the downstream transcription factors ABRE (ABA-responsive element binding) proteins or AREB/ABF (ABRE-binding factors) [11,12,13,14,15,16,17]. The phosphorylated transcription factors activate or inhibit ABA-responsive genes through the DNA-binding element on their promoter regions [18,19]. 

PP2Cs, which belong to the largest protein phosphatase family in plants, are the core components of ABA signaling. The following PP2Cs have been identified: ABI1 (ABA-INSENSITIVE 1), ABI2, HAB1 (ABA-hypersensitive 1), HAB2, AHG1 (ABA HYPERSENSITIVE GERMINATION 1), and PP2CA/AHG3 [20,21]. A recent study showed that AtPP2CF1 (PP2C ISOFORM), an ABI1-interacting protein in *Arabidopsis*, is insensitive to ABA during seed germination and under drought stress [22]. AtAHG1, which encodes a protein phosphatase 2C in mutant *ahg1*, is more hypersensitive to ABA, salinity, mannitol, glucose, and sucrose at the seed germination and post-development stages than its analogue AtAHG3 in mutant *ahg3* [23]. In maize, *ZmPP2C-A*s are dramatically induced by multiple stresses. *ZmPP2C-A2* and *ZmPP2C-A6* overexpression causes hyposensitivity to exogenous ABA and NaCl treatments at the germination stage and negatively regulates the drought stress response when exposed to drought stress [24]. In wheat, *TaPP2C-a10* interacts with the dormancy genes *TaDOG1L1* (DELAY OF GERMINATION 1) and *TaDOG1L4* (DELAY OF GERMINATION 4) and decreases tolerance to drought stress in transgenic *Arabidopsis* [25]. Although 128 MdPP2Cs have been identified [26], their functions are still unclear.

In this study, *MdPP2C24/37*, two group A PP2C genes in apple (*huashuo*), were cloned and their functions in ABA signaling were identified. Under drought stress, signal transduction consists of the osmotic homeostasis signaling pathway [27,28]. Therefore, this research studied osmotic stress induced by mannitol. RT-qPCR analysis revealed that *MdPP2C/37* were induced by ABA or mannitol. Compared with WT plants, *MdPP2C24/37* overexpression lines in *Arabidopsis* were hyposensitive to ABA or mannitol at the seed germination and seedling establishment stages. Transcriptional analysis showed that stress-mediated genes, represented by *DREB2A* and *KIN2*, were upregulated in the *MdPP2C24/37* overexpression lines under control conditions. Meanwhile, *ABF4* and *RD29A* were only upregulated after plants were treated with exogenous ABA or mannitol. When exposed to drought stress, all of the transgenic plants exhibited inhibited stomatal closure, significantly higher water loss rates, and lower survival rates than WT plants. Under drought stress, MDA and H_2_O_2_ levels accumulated while chlorophyll levels decreased more rapidly in the overexpression plants than in WT plants. Furthermore, MdPP2C24/37 interacted with MdPYL2/12 in vitro and vivo, respectively. Taken together, these results indicate that MdPP2C24/37 act as negative regulators in ABA signaling and are involved in ABA-mediated drought resistance.

## 2. Results

### 2.1. Structure and Homology Characterization of MdPP2C24/37

**Figure 1:**
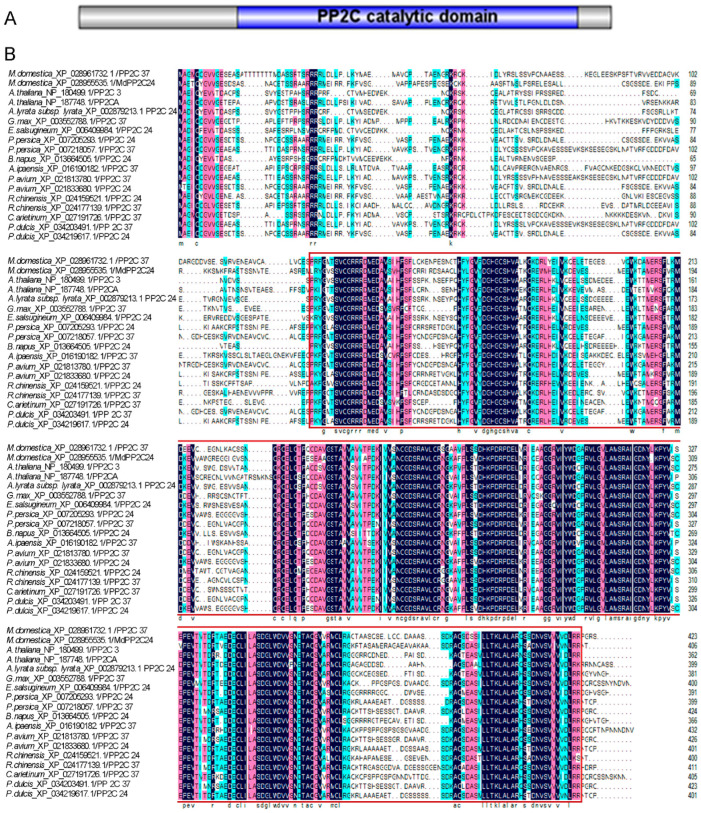
The amino acid sequence analysis of MdPP2C24/37. (**A**) Both MdPP2C24/37 were predicted to have PP2C catalytic domains. (**B**) Sequence analysis indicated that MdPP2C24/37 are highly conserved proteins analogous to proteins in *Arabidopsis thaliana*, *Arabidopsis lyrata* subsp. *Lyrata*, *Arachis ipaensis*, *Brassica napus*, *Cicer arietinum*, *Eutrema salsugineum*, *Glycine max*, *Rosa chinensis*, *Prunus persica*, *Prunus avium,* and *Prunus dulcis*.

In apple, *MdPP2C24 (LOC103407751, MDP0000883465)* encodes a 408-amino acid protein on the third chromosome, while *MdPP2C37 (LOC114826111, MDP0000203818)* encodes a 424-amino acid protein on the seventh chromosome. In this study, based on the NCBI website, both MdPP2C24/37 were predicted to possess conserved PP2C catalytic domains (Figure 1A). Using MEGA 7, a phylogenetic tree was generated according to the highly conserved PP2C homologous protein sequences (Figure 1B, Supplementary Figure S1). In addition, the phylogenetic tree analysis revealed that MdPP2C24 shared 56.69% identity with AtPP2C3, and MdPP2C37 shared 60.55% identity with AtPP2CA (Figure 1B). These results indicated that *MdPP2C24/37* belong to the conserved PP2C gene family in plants.

### 2.2. MdPP2C24/37 Genes Are Induced by ABA Treatment or Mannitol Stress in Apple

**Figure 2:**
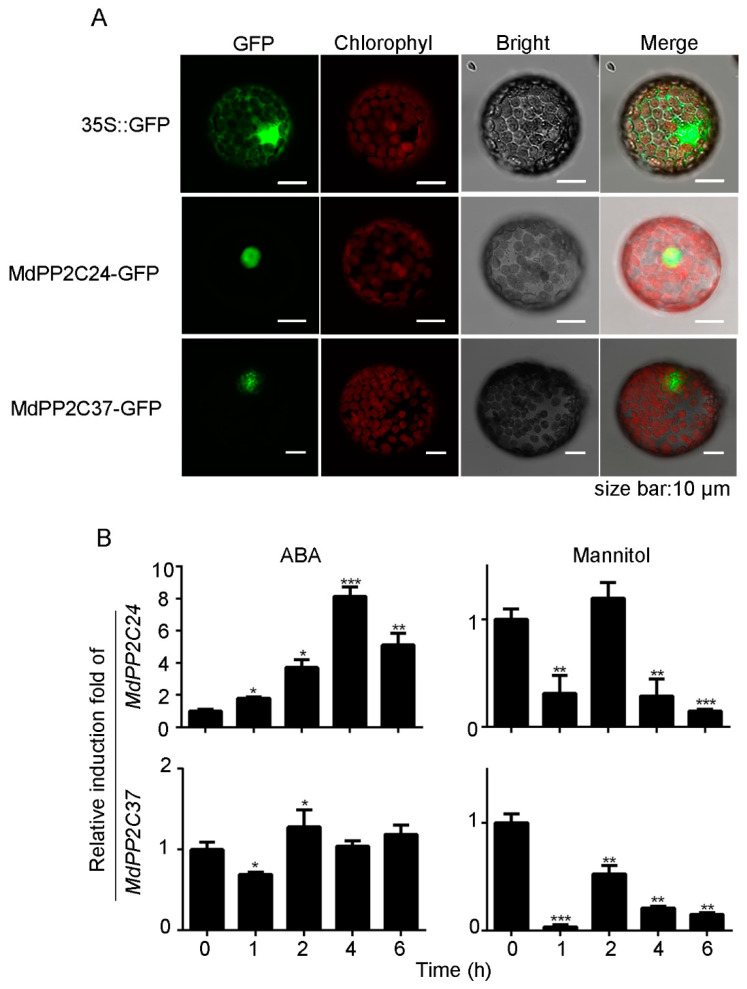
Nucleus-located genes *MdPP2C24/37* were induced by ABA or mannitol. (**A**) Subcellular localization of MdPP2C24/37-GFP fusion proteins in tobacco protoplasts. *35S*::GFP alone or MdPP2C24/37-GFP correspond to chlorophyll and bright field images, respectively, and the superposition of fluorescent illumination, chlorophyll, and bright field images is shown. (**B**) Real-time qPCR analysis of *MdPP2C24/37* expression in apple leaves. Total RNA was isolated from the ABA- or mannitol-treated apple leaves and used for real-time qPCR. Results represent mean ± SE from three independent experiments, with similar results obtained. Values were significantly different from WT at * *p* < 0.05, ** *p* < 0.01 or *** *p* < 0.001.

To identify the cellular localization of MdPP2C24 and MdPP2C37, we performed in vivo transient expression analysis in tobacco protoplasts. *35S*::MdPP2C24-GFP, *35S*::MdPP2C37-GFP, and *35S*::GFP constructs were transformed into tobacco leaf protoplasts using a polyethylene glycol (PEG)-mediated method [29]. The fluorescence signal of MdPP2C24/37-GFP fusion proteins was observed in the nucleus, indicating that MdPP2C24/37 are nuclear proteins (Figure 2A).

To preliminarily study the *MdPP2C24/37* response to drought stress in apple, we performed a 50 μM ABA or 200 mM mannitol treatment on apple leaves (Figure 2B). The transcriptional analysis results using qRT-PCR supported the possibility that *MdPP2C24/37* are involved in ABA or osmotic signaling in apple.

### 2.3. MdPP2C24/37 Regulate ABA Response in Germination and Seedling Establishment through Stress-Responsive Genes in Arabidopsis

**Figure 3:**
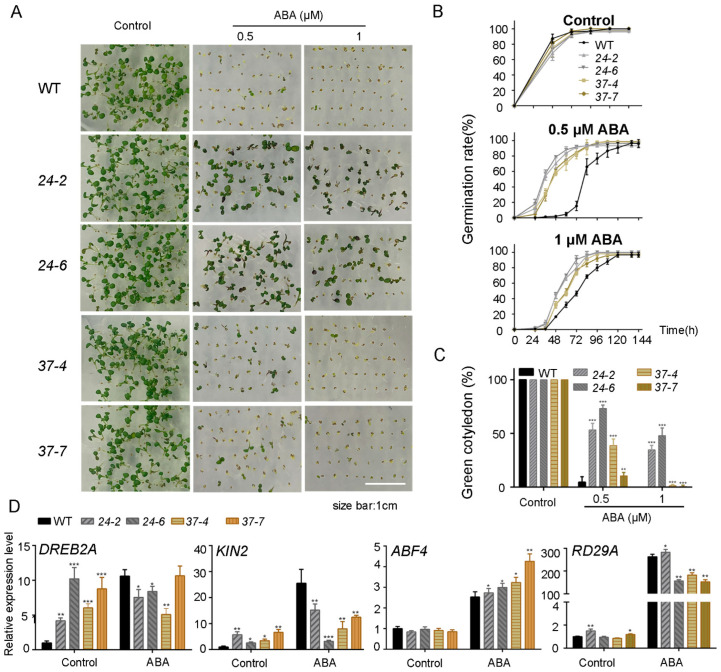
Phenotypic analysis of overexpression of *MdPP2C24/37* in *Arabidopsis* revealed a hyposensitive phenotype to ABA at the seed germination stage. (**A**) Representative images of seed germination. (**B**) Statistical analysis of seed germination rate. (**C**) Cotyledon greening rate in WT and transgenic lines 6 days after seeds were sown in MS medium supplemented with 0, 0.5, and 1 μM ABA. Results represent the mean ± SD from 3 independent experiments. (**D**) Real-time qPCR analysis of stress-responsive gene expression changes in *MdPP2C24/37* overexpression lines in *Arabidopsis.* The expression levels were based on total RNA extracted from WT and *MdPP2C24/37* overexpression lines in liquid MS medium, or liquid MS medium supplemented with 50 μM ABA for 3 h. Results represent the mean ± SE from 3 independent experiments. The expression levels are presented as relative units, with levels under control conditions taken as 1. All experiments were replicated three times with similar results. Values were significantly different from WT at * *p* < 0.05, ** *p* < 0.01 or *** *p* < 0.001.

To study the gene function of *MdPP2C24/37* properly, we generated *MdPP2C24/37* overexpression lines in *Arabidopsis* driven by the cauliflower mosaic virus (CaMV) 35S promoter. PCR analysis of DNA levels and real-time qPCR analysis of RNA transcript levels confirmed *MdPP2C24/37* overexpression in these lines (Supplementary Figure S2). Three independent overexpression lines were obtained for each MdPP2C. Then, overexpression lines *MdPP2C24 (24-2, 24-6)* and *MdPP2C37 (37-4, 37-7)* were chosen as representative lines. To study the roles of MdPP2Cs in the ABA response, we germinated the plants in MS media containing different concentrations of ABA. After treatment with 0.5 or 1 μM ABA, the seed germination rates of the *MdPP2C24/37* overexpression lines were significantly higher than those of WT (Figure 3A,B). In addition, the *MdPP2C24/37* overexpression lines exhibited markedly higher cotyledon greening rates under the 0.5 μM ABA treatment than WT, including over 50% in the *MdPP2C24* overexpression lines and over 20% in the *MdPP2C37* overexpression lines. In contrast, only a 4% cotyledon greening rate was recorded in the wild type plants. The same cotyledon greening rate trend was observed after treatment with 1 μM ABA; namely almost 40% in the *MdPP2C24* overexpression lines and 2% in the *MdPP2C37* overexpression lines, whereas no cotyledon greening was observed in WT (Figure 3C). In these results, no significant difference was observed among plants under control conditions (Figure 3A–C, Supplemental Figure S3). Next, the 7-day-old seedlings were treated with liquid MS medium to which 50 μM ABA was added. A qPCR assay was used to detect the stress-responsive gene levels of *DREB2A*, *KIN2*, *ABF4,* and *RD29A*. The results showed that transcript levels of *DREB2A* and *KIN2* were notably increased in the *MdPP2C24/37* overexpression lines compared to WT under control conditions. After exogenous ABA treatment, transcript levels of *DREB2A* and *KIN2* were almost unchanged in the *MdPP2C24/37* overexpression lines; however, they were significantly increased in WT. Moreover, transcript levels of *ABF4* and *RD29A* were upregulated after ABA treatment in all plants (Figure 3D). These results indicated that MdPP2C24/37 decreased ABA sensitivity at the germination and seedling stages in *Arabidopsis*.

### 2.4. Overexpression of MdPP2C24/37 Modulate Drought Tolerance

**Figure 4:**
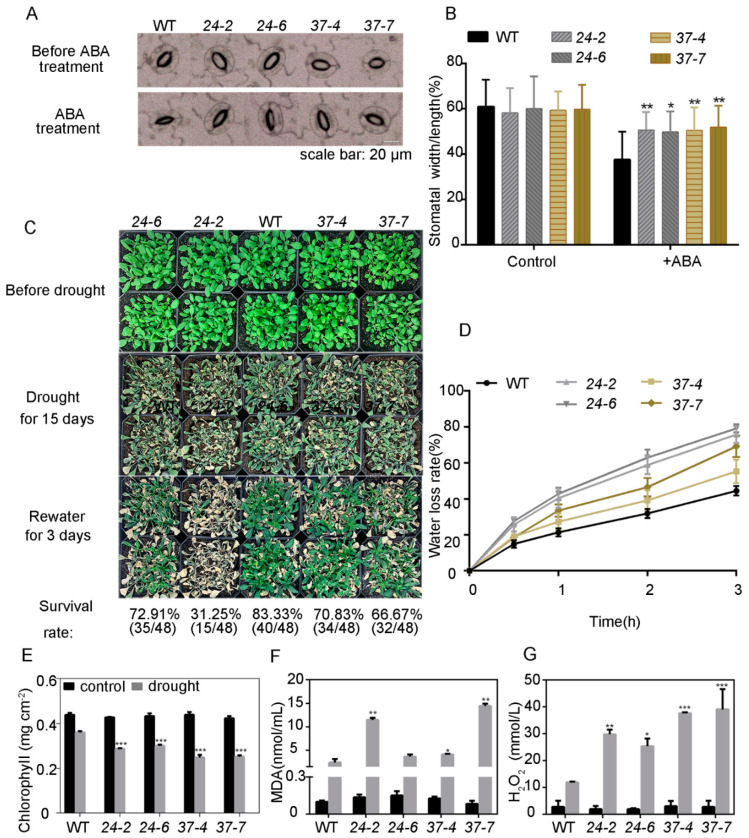
The *MdPP2C24/37* overexpression lines in *Arabidopsis* displayed decreased drought stress tolerance compared to WT. (**A**,**B**) Stomatal aperture assay induced by ABA of *MdPP2C24/37* overexpression lines in *Arabidopsis*. (**A**) Representative images of stomatal aperture and (**B**) statistical analysis of stomatal aperture width/length. Values represent the mean ± SD from three independent experiments; n = 80 per experiment. (**C**) Drought tolerance assay of WT and *MdPP2C24/37* overexpression lines. Three-week-old plants were exposed to drought stress for 15 days and then rewatered for three days. Values represent the mean ± SD from three independent experiments; n = 48 per experiment. (**D**) Water loss rates during 3 h period in detached leaves of WT and *MdPP2C24/37* overexpression lines. Values represent the mean ± SD of five individual plants per genotype. (**E**–**G**) Plants of all genotypes subjected to drought through withholding of water for 12 days. (**E**) Chlorophyll (**F**) MDA, and (**G**) H_2_O_2_ levels were measured. Results represent the mean ± SD from three independent experiments, with similar results obtained. Values were significantly different from WT at * *p* < 0.05, ** *p* < 0.01 or *** *p* < 0.001.

Next, the relationship between *MdPP2C24/37* and ABA-dependent drought stress response was studied. A stomatal aperture assay revealed that the *MdPP2C24/37* overexpression lines were insensitive to ABA compared with WT (Figure 4A,B). Under normal conditions, however, no differences were observed among these plants. However, higher water loss rates were observed in the detached leaves of the *MdPP2C24/37* overexpression lines at the indicated time points (Figure 4D). Furthermore, when exposed to drought stress for 15 days, fewer *MdPP2C24/37* overexpression lines survived than WT (Figure 4C). 

In this study, no significant differences were observed for chlorophyll, MDA, and H_2_O_2_ levels among plants under control conditions. However, under drought stress, chlorophyll levels decreased more rapidly in all *MdPP2C24/37* overexpression lines than in WT (Figure 4E). Further, MDA and H_2_O_2_ accumulation was significantly higher in *MdPP2C24/37* overexpression lines than in WT (Figure 4F,G). The drought stress method used in this study involved withholding water for 12 days. In general, the results presented above indicated that the *MdPP2C24/37* overexpression lines showed decreased resistance ability to ABA-mediated drought stress in adult *Arabidopsis*. 

### 2.5. MdPP2C24/37 Involved in Response to Osmotic Stress through Stress-Responsive Genes in Arabidopsis

**Figure 5:**
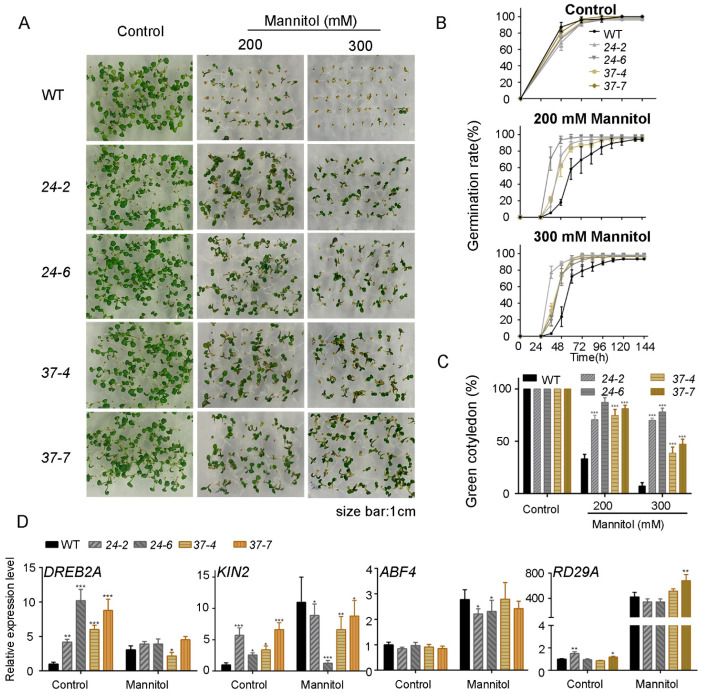
Phenotypic analysis of *MdPP2C24/37* overexpression lines in *Arabidopsis*, which caused a hyposensitive phenotype to mannitol at the seed germination stage. (**A**) Representative images of seed germination. (**B**) Statistical analysis of seed germination rate. (**C**) Cotyledon greening rates of WT and transgenic lines 6 days after seeds were sown in MS medium supplemented with 0, 200, and 300 mM mannitol. Results represent the mean ± SD from 3 independent experiments. (**D**) Real-time qPCR analysis of stress-responsive gene expression changes in the *MdPP2C24/37* overexpression lines in *Arabidopsis.* The expression levels were based on total RNA extracted from WT and transgenic *Arabidopsis* in liquid MS medium, or liquid MS medium supplemented with 200 Mm mannitol for 3 h. The expression levels are presented as relative units, with levels under control conditions taken as 1. Results represent the mean ± SE from 3 independent experiments, with similar results obtained. Values were significantly different from WT at * *p* < 0.05, ** *p* < 0.01 or *** *p* < 0.001.

Next, the *MdPP2C24/37* overexpression lines were studied in response to osmotic stress. The germination and cotyledon greening rates of the *MdPP2C24/37* overexpression lines were higher than those of WT under 200 or 300 mM mannitol treatment (Figure 5A,B). All of the *MdPP2C24/37* overexpression lines displayed over 80% cotyledon greening rates, while the wild type plants only showed 35% under the 200 mM mannitol treatment. Further, the *MdPP2C24* overexpression lines displayed almost 70% cotyledon greening rates, the *MdPP2C37* overexpression lines displayed 40%, and WT only displayed 7% in MS medium supplemented with 300 mM mannitol (Figure 5C). In these results, no significant difference was observed among plants under control conditions (Figure 5A–C, Supplementary Figure S3). Subsequently, the 7-day-old seedlings were treated with liquid MS medium containing 200 μM mannitol. A real-time qPCR assay was used to detect the stress-responsive gene levels of *DREB2A*, *KIN2*, *ABF4,* and *RD29A*. *DREB2A* and *KIN2* expression levels in the *MdPP2C24/37* overexpression lines were much higher than those in WT under control conditions. Under exogenous mannitol treatment, transcript levels of *DREB2A* and *KIN2* were almost unchanged in the *MdPP2C24/37* overexpression lines; however, most transcript levels were significantly increased in WT. Further, transcript levels of *ABF4* and *RD29A* were upregulated after mannitol treatment in all plants (Figure 5D). To summarize, MdPP2C24/37 decreased osmotic sensitivity at the germination and seedling stages in *Arabidopsis*.

### 2.6. Both MdPP2C24/37 Interacted with MdPYL2/12 In Vitro and Vivo

**Figure 6:**
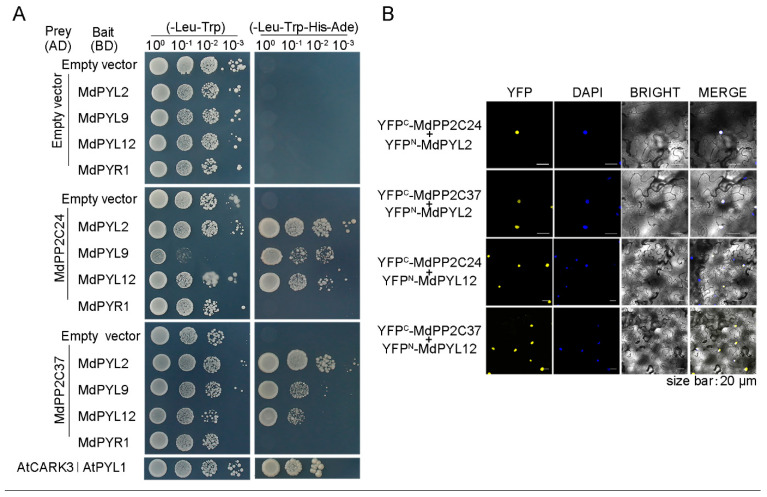
MdPP2C24/37 interacted with MdPYL2/12 in vitro and vivo. (**A**) MdPP2C24/37 interacted with MdPYL2/9/12 in the yeast two-hybrid assay. AD–MdPP2C24/37 fusion prey vectors were co-transformed with BD–MdPYL2/9/12/PYR1 fusion bait vectors into yeast cells. Positive interactions were indicated by the ability of cells to grow on SD/−Leu/−Trp/−His/−Ade dropout medium. Empty AD prey vector and BD bait vectors were used as negative controls. (**B**) MdPP2C24/37 interacted with MdPYL2/12 in the bimolecular fluorescence complementation (BiFC) assay, showing fluorescence in nuclei of tobacco leaf epidermal cells. The C-terminus part of YFP was fused to MdPP2C24/37, and the N-terminus part of YFP was fused to MdPYL2/12. All experiments were replicated three times, with the same results obtained.

In this study, the coding sequences of MdPP2C24/37 were used as prey and several possible MdPYLs/PYR1 were used as bait in a yeast two-hybrid assay. Under the four-minus selection medium (SD/-Leu/-Trp/-His/-Ade) growth conditions, MdPYR1 was unable to interact with MdPP2Cs, while MdPYL2/9/12 interacted with MdPP2C24/37 in vitro (Figure 6A). 

To further confirm the associations of MdPP2C24/37 and MdPYLs/PYR1 in vivo, we performed a BiFC assay. Fluorescence signals were observed only when MdPP2C24/37 and MdPYL2/12 interacted (Figure 6B). MdPP2C24/37 and MdPYL2/12 interacted in the nucleus, which was stained with the nucleic acid dye DAPI. The interaction between YFP^C^-AtCARK3 + YFP^N^-AtPYL1 was adopted as a positive control, while interactions between YFP^C^-MdPP2C24/37 + YFP^N^ and YFP^C^ + YFP^N^-MdPYL2/12 were adopted as negative controls (Supplementary Figure S4A). However, no fluorescence signal was detected between MdPP2C24/37 and MdPYL9/PYR1 (Supplementary Figure S4B). These results indicated that both MdPP2C24/37 interacted with MdPYL2/12 in vitro and in vivo. 

## 3. Discussion

Adverse environmental cues severely affect plant growth and development. To deal with stress, plants have developed a series of strategies, such as ABA signal conduction [14,30,31,32]. PP2Cs, important signal transductors in ABA signaling, have been reported in species such as *Arabidopsis* [33,34], maize [24], soybean [35], *Pyrus bretschneideri* [26], *Brassica rapa* [36], wheat [37], and *Gossypium hirsutum* [38]. However, the PP2C gene family has not been studied widely in the *Rosaceae* family, represented by apple. In this study, MdPP2C24/37, having highly conserved PP2C catalytic domains, were identified through sequence alignment (Figure 1, Supplementary Figure S1) according to bioinformatics analysis [26], a method which is consistent with previous studies [39]. 

Considerable evidence indicates that PP2C localization is required for different functions. In *Arabidopsis*, plasma membrane-localized members of PP2Cs, namely PP2C.D2, PP2C.D5, and PP2C.D6, are major regulators of cell expansion through their physical interaction with SAUR19 and PM H^+^-ATPases, and they inhibit cell expansion by dephosphorylating the penultimate threonine of PM H^+^-ATPases [40]. AtABI1, a nuclear protein, is essential to confer insensitivity towards ABA [41]. In this study, MdPP2C24/37 were nucleus-located and induced by ABA (Figure 2). The results indicated that both MdPP2Cs are probably involved in nuclear signal transduction, which prompted us to analyze their functions in ABA responsiveness. Further results revealed that the expression of *MdPP2C24/37* in *Arabidopsis* led to plant insensitivity to ABA during seed germination and seedling establishment and to ABA-induced drought stress (Figure 3 and Figure 4, Supplementary Figure S3). Previous reports revealed that PP2Cs are involved in ABA signaling through their interaction with ABA receptors PYR/PYLs [32,42,43,44,45]. However, whether MdPP2Cs associate with MdPYLs/PYR1 in apple is not clear. In this study, MdPP2C24/37 were found to interact with ABA receptors MdPYL2/12 in the nucleus through a yeast two-hybrid assay and BiFC assay (Figure 6). The results proved that MdPP2C24/37 are negative regulators, as typical PP2Cs, in ABA signaling. However, MdPYL9, a homologue to PYL9 in *Arabidopsis*, *Zea mays,* and *M. domestica* [46,47,48], was not observed to interact with MdPP2C24/37 in vivo (Figure 6, Supplementary Figure S4). In summary, these results suggest that MdPP2C24/37 interact with MdPYL2/12, specifically in the nucleus, and play negative roles in ABA signaling. 

In higher plants, ABA signaling can be triggered by multiple stresses, such as drought and osmotic stress [27]. In addition, some PP2Cs play pivotal roles in osmotic stress [49]. For instance, *ZmPP2C-As* in maize has been found to be dramatically induced by osmotic stress, resulting in a higher seed germination rate under osmotic stress and higher death rate when plants are exposed to drought stress [24]. *OsPP108* and *OsSIPP2C1* in rice, which are upregulated by high salt, exogenous ABA, and drought treatment, act as negative regulators in ABA signaling [50,51]. In this study, mannitol was used to reveal the roles played by MdPP2C24/37 in the relationship between ABA and osmotic stress signaling. The results indicated that transcript levels of *MdPP2C24/37* were induced by ABA or mannitol (Figure 2). The *MdPP2C24/37* overexpression lines were hyposensitive at the germination and seedling establishment stages under ABA or mannitol treatment in *Arabidopsis* (Figure 3 and Figure 5, Supplementary Figure S3). Further, stress-related genes, namely *DREB2A*, *KIN2*, *ABF4,* and *RD29A*, exhibited almost the same expression tendencies under ABA or mannitol treatment in all *MdPP2C24/37* overexpression plants (Figure 3 and Figure 5). Plants induce stomatal movement through ABA signaling [32] and closely guard cells to maintain water balance when they are exposed to drought stress [52]. In this study, all of the *MdPP2C24/37* overexpression lines displayed inhibited stomatal closure in response to exogenous ABA treatment (Figure 4). Moreover, osmotic stress, which accompanies water deficit, can be regulated through ABA-dependent signaling [28,53]. Overall, this study comprehensively reported the roles of MdPP2C24/37 at different stages. At the seed germination and seedling establishment stages, *MdPP2C24/37* overexpression plants were hyposensitive to both ABA and mannitol. At the adult stage, overexpression of *MdPP2C24/37* in *Arabidopsis* led to a lower survival rate through its inhibition of stomatal closure (Figure 3, Figure 4 and Figure 5). These results are consistent with those found for *PtNF-YA9* (NUCLEAR FACTOR Y9) in *Populus trichocarpa* [54]. In summary, these observations demonstrate that MdPP2C24/37 participate in multiple signaling pathways at different growth stages and function as connecting links in response to ABA signaling and osmotic stress.

Current evidence suggests that redundancies in PP2Cs create major obstacles to studying the functions of PP2Cs genes using genetic approaches [55]. For instance, ABI1 and ABI2 phosphatases play overlapping roles in controlling ABA actions [10]. It is noteworthy that both MdPP2C24/37 have highly conserved PP2C catalytic domains but share only 54% homologous similarity (Figure 1). This analysis suggests that MdPP2C24/37 may have different functions, as well as redundant functions, in ABA signaling in apple. That is, the functions of MdPP2C24/37 may diverge at some point in response to ABA and osmotic stress, and the functions of their potential targets may be different from those of MdPYL2/12 (Figure 6). Therefore, these synergistic effects further suggest that MdPP2C24/37 may participate in other signaling pathways.

## 4. Materials and Methods

### 4.1. Plant Materials and Growth Conditions

Subcultured ‘*huashuo*’ apple [3] shoots were propagated in MS medium supplemented with 2.0 mg L^−1^ TDZ (thidiazuron, Cat. #P6186, Sigma-Aldrich, America), 0.1 mg L^−1^ IBA (3-indole butyric acid, Sigma-Aldrich), 30 g L^−1^ sucrose, and 7 g L^−1^ agar (pH 5.8). Seeds of *Arabidopsis thaliana* (WT, Col-0) and *Nicotiana benthamiana* were sterilized by soaking them in 20% NaClO (*v*/*v*) for 15 min, and they were washed 6 times to remove residual NaClO solution. Sterilized seeds were sown in MS medium containing 2% sucrose and 0.8% agar (pH 5.8), and vernalized at 4 °C for 3 days. All plants were cultivated at 22 °C under long day conditions with a cycle of 16 h light/8 h dark) and 180 μmol m^−2^ s^−1^.

### 4.2. Real-Time qPCR Analysis

Total RNA of fresh leaves from four-week-old subculture apple shoots were extracted using the CTAB assay [56]. RNA of *A. thaliana* was isolated from 7-day-old seedlings using RNAiso Plus (Cat. #9109, Takara, Japan). Reverse transcription was performed using the cDNA Synthesis Kit (Cat. #RR047A, Takara, Japan). Real-Time qPCR was performed using SYBR Premix Ex Taq (Cat. # RR430A, Takara, Japan). *ACTIN2 (At3g18780)* was used as an internal control for *Arabidopsis*, while *MdEF-1α (LOC103443462)* was used as an internal control for apple. All gene-specific primer pairs are listed in Supplementary Table S1.

### 4.3. Amino Acid Sequence Alignment and Phylogenetic Analysis

The predicted amino acids of MdPP2C24/37 and their homologous proteins were obtained from NCBI (https://www.ncbi.nlm.nih.gov/, accessed on 1 January 2022) and JGI (https://phytozome.jgi.doe.gov/pz/portal.html#, accessed on 1 January 2022). The homologous proteins of MdPP2C24/37 in *A. thaliana*, *Arabidopsis lyrata* subsp. *Lyrata*, *Arachis ipaensis*, *Brassica napus*, *Cicer arietinum*, *Eutrema salsugineum*, *Glycine max*, *Rosa chinensis*, *Prunus persica*, *Prunus avium,* and *Prunus dulcis* were obtained using the NCBI BLAST tool. The protein domain prediction map was created using IBS1.0.2 software (http://ibs.biocuckoo.org/, accessed on 1 January 2022). The phylogenetic tree was generated using the Neighbor-Joining method in MEGA 7 software, with 500 replicated bootstrap values used at each node [57]. Sequence alignment was performed using DNAMAN9 (http://www.lynnon.com/, accessed on 1 January 2022).

### 4.4. Transient Expression Assay and Transgenic Arabidopsis Constructs

To achieve transient expression in tobacco mesophyll cell protoplasts, the MdPP2C24/37 CDSs were cloned into the *p*BI221-*eGFP* vector. Analysis of transient expression in protoplasts was performed as previously described [29].

To generate transgenic *Arabidopsis*, MdPP2C24/37 CDSs were cloned into the *p*Cambia2300 vector. The plasmids were introduced into *Arabidopsis* Col-0 by *Agrobacterium tumefaciens* (GV3101)-mediated transformation. The floral dip method was adopted in this study [58]. The seeds of the transgenic plants were screened in MS medium supplemented with 35 mg/L kanamycin. The T3 homozygous seeds were used for phenotypic analysis. The primers used in this assay are listed in Supplementary Table S1.

### 4.5. Phenotype Analysis

For germination and seedling establishment assays, approximately 150 sterilized seeds of each line were sown in MS medium with various concentrations of ABA (Cat. #90769, Sigma-Aldrich, America) or mannitol (Cat. #M8140, Solarbio, China). The germination rate was calculated every 12 h 6 days after sowing. The cotyledon greening rates were calculated on the 6th day after sowing. 

For the seedling establishment assay, seeds grew vertically in 1/2 MS medium for 7 days with 0 or 0.5 μM ABA or 300 mM mannitol. The root lengths were measured using ImageJ software (https://imagej.en.softonic.com/, accessed on 1 January 2020).

For the stomatal aperture assay, rosette leaves of 3-week-old plants were detached and floated in stomatal opening solution (5 mM KCl, 10 mM MES (2- (N-morpholino) ethanesulfonic acid), 50 μM CaCl2, pH 6.15) in the dark for 2 h, then exposed to continuous light for 2 h, followed by the addition of 10 μM (±) ABA. In order to estimate ABA-induced stomatal closure, apertures were recorded on epidermal strips after 2 h incubation. Width/length ratios were measured and counted using ImageJ software (v1.8.0, National Institutes of Health, America).

For the water loss assay, rosette leaves of 3-week-old *Arabidopsis* plants were detached. The water loss rate was monitored at indicated times. For drought stress analysis, 3-week-old plants were not watered for 15 days and then rewatered. The survival rate was calculated.

### 4.6. Determination of Drought-Responsive Physiological Indices

The 3-week-old *Arabidopsis* plants were subjected to normal conditions or exposure to drought stress. Then, leaves were collected for chlorophyll, malondialdehyde (MDA), and H_2_O_2_ content determination according to a previously described protocol [59].

### 4.7. Yeast Two-Hybrid Assay

The yeast two-hybrid assay was performed according to a previously described protocol [60]. *MdPYL2 (LOC103440595, MDP0000147358), MdPYL9 (LOC103413926, MDP0000284624*)*, MdPYL12 (LOC103434883, MDP0000132875),* and *MdPYR1 (LOC103436894*, *MDP0000125850*) were investigated in this study. The CDSs of MdPP2C24/37 were cloned into the pGADT7 vector (AD). The CDSs of MdPYL2, MdPYL9, PYR1, and MdPYL12 were cloned into the pGBKT7 vector (BD). 

The AD-MdPP2C24/37 and BD-MdPYL2/9/12/PYR1 plasmids were co-transformed into yeast AH109 cells. The yeast cells were plated in SD/-Leu/-Trp medium for 2–3 days, and in SD/-Leu/-Trp/-His/-Ade medium for 5–8 days. AD-AtCARK3+BD-AtPYL1 was used as the positive control [61]. 

### 4.8. Bimolecular Fluorescence Complementation (BiFC) Assay

The BiFC assay was performed according to a previously described protocol [62]. The BiFC vectors pSPYNE (YFP^N^)/pSPYCE (YFP^C^) harbor either the N-terminus or C-terminus of eYFP. YFP^N^ was fused to the C termini of MdPYL2/9/12, and PYR1 and YFP^C^ were fused to the C termini of MdPP2C24/37. The vectors were transformed into *A. tumefaciens* GV3101. Pairwise construct combinations were transiently expressed in *N. benthamiana* epidermal cells. The previously described YFPC-AtCARK3 and YFPN-AtPYL1 constructs were used as positive controls [34]. The fluorescence signal of YFP was detected after infiltration for 3 days using an Olympus optical microscope at an excitation wavelength of 560 nm. The primers used for these constructs are listed in Supplementary Table S1.

### 4.9. Statistical Analysis

Data are represented as the mean ± SD and ± SEM. Statistical analysis was performed using Student’s *t*-test. Values were significantly different from WT at * *p* < 0.05, ** *p* < 0.01 or *** *p* < 0.001.

## 5. Conclusions

In conclusion, this study demonstrated that MdPP2C24/37, isolated from ‘*huashuo*’, are nucleus-located and negatively regulate seed germination, seedling establishment, stomatal aperture, and stress-related gene expression in response to drought stress. The interactions between MdPP2C24/37 and ABA receptors MdPYL2/12 provide new a direction for studying the ABA transduction mechanism in apple. Overall, these results provide evidence that *MdPP2C2C24/37* respond to ABA and osmotic stress, thus enriching the ABA signal regulation network in apple. 

## Data Availability

Not applicable.

## Supplementary Materials

The following supporting information can be downloaded at: https://www.mdpi.com/article/10.3390/ijms232214375/s1.
