# Supplementary material for: MdPP2C24/37, Protein Phosphatase Type 2Cs from Apple, Interact with MdPYL2/12 to Negatively Regulate ABA Signaling in Transgenic Arabidopsis
Source: Int J Mol Sci. 2022 Nov 19;23(22):14375. doi: 10.3390/ijms232214375 (PMC9696740; doi:10.3390/ijms232214375)
Supplement: Supplementary file 1 [file ijms-23-14375-s001.zip › ijms-1988420-supplementary.pdf]

Supplementary Materials

Figure S1. The phylogenetic tree analysis of MdPP2C24/37.

Figure S2. Identification of the *MdPP2C24/37* overexpression lines in *Arabidopsis.*

Figure S3. Phenotypic analysis of overexpression of *MdPP2C24/37* in *Arabidopsis* found that it has a hyposensitive phenotype to ABA or mannitol at seedling establishment stage.

Figure S4. Positive control and negative results using bimolecular fluorescence complementation (BiFC) assay of this study.

Supplemental Table S1 List of Primers Used in This Study.

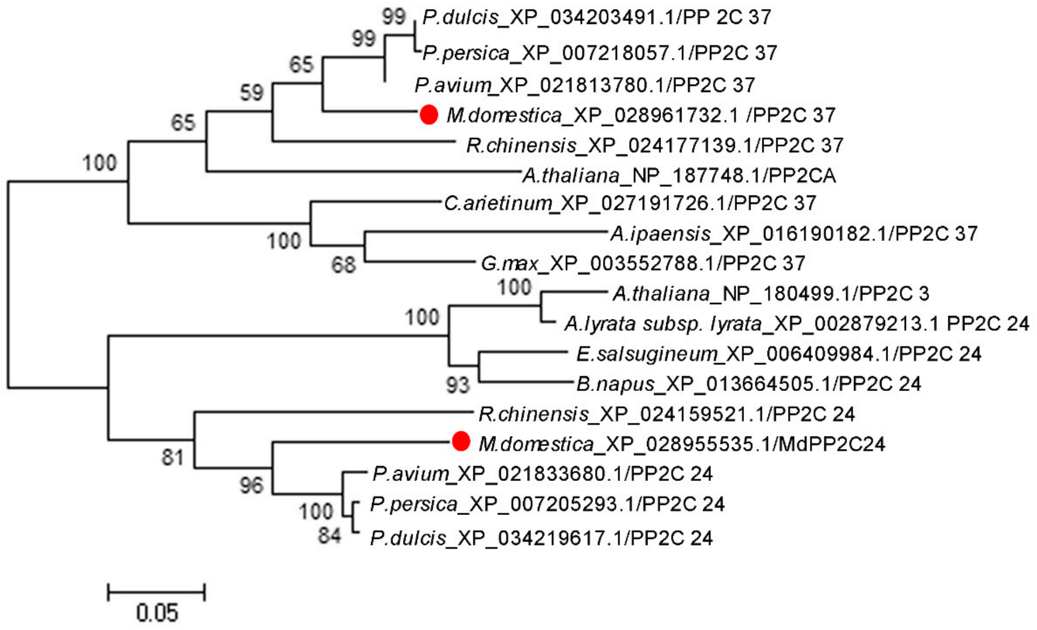

Figure S1. The phylogenetic tree analysis of MdPP2C24/37. The phylogenetic tree of PP2Cs was generated based on the alignment of the full-length PP2Cs protein sequence in several species including *Arabidopsis thaliana*, *Arabidopsis lyrata subsp. Lyrata*, *Arachis ipaensis*, *Brassica napus*, *Cicer arietinum, Eutrema salsugineum*, *Glycine max*, *Rosa chinensis*, *Prunus persica*, *Prunus avium* and *Prunus dulcis*. Bootstrap value from 500 replicates indicated at each node, using MEGA software version 7. The scale bar represents the branch length.

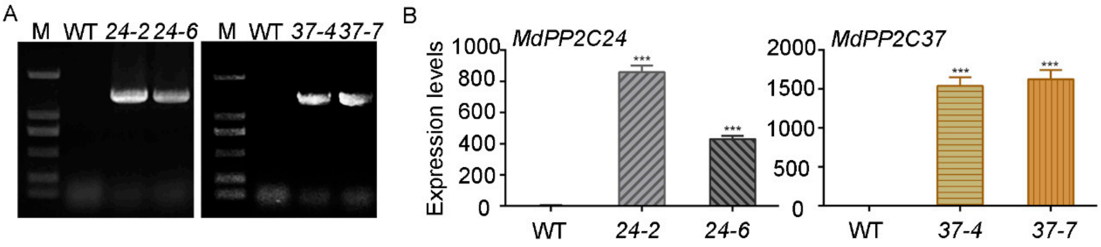

Figure S2. Identification of the *MdPP2C24/37* overexpression lines in *Arabidopsis.* (A-B) RT-PCR analysis on the DNA (A) and RNA (B) levels proved that the *MdPP2C24/37* overexpressed in *Arabidopsis* successfully. The expression levels are presented as relative units with the levels of control being taken as 1. Results are means ± SE from 3 independent experiments. All experiments were replicated three times, with the same results obtained.

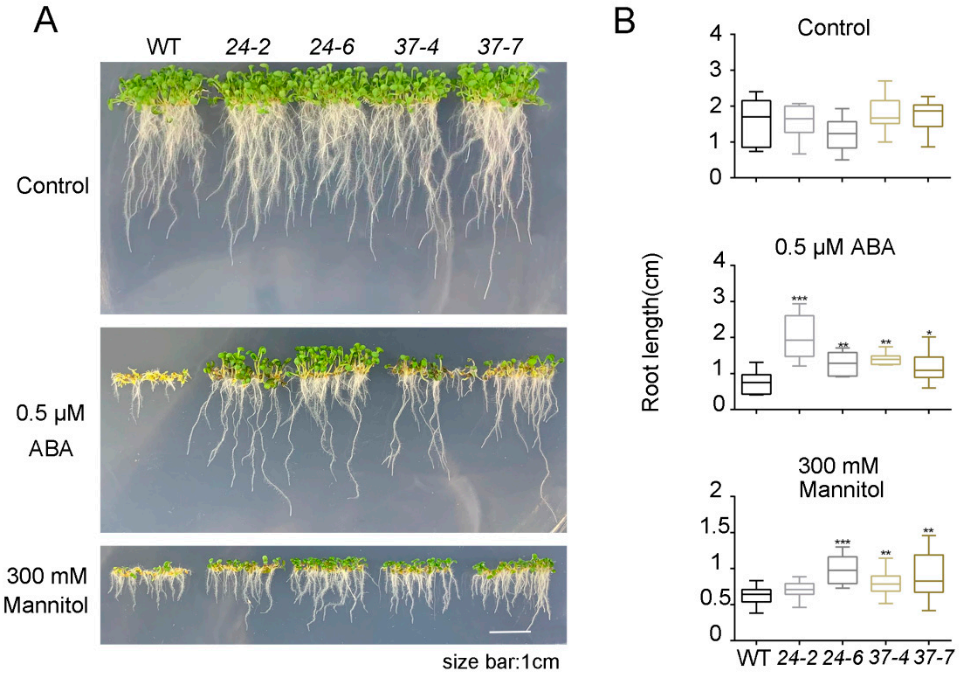

Figure S3. Phenotypic analysis of overexpression of *MdPP2C24/37* in *Arabidopsis* found that it has a hyposensitive phenotype to ABA or mannitol at seedling establishment stage. (A) Representative images of morphology of roots length, (B) roots length statistics of WT and the *MdPP2C24/37* overexpression seeds were cultivated 7 d after seeds were sowed in 1/2 MS medium vertically with medium supplemented with 0, 0.5 μM ABA or 300 mM mannitol. Results are means ± SD from 3 independent experiments. All experiments were replicated three times, with the same results obtained.

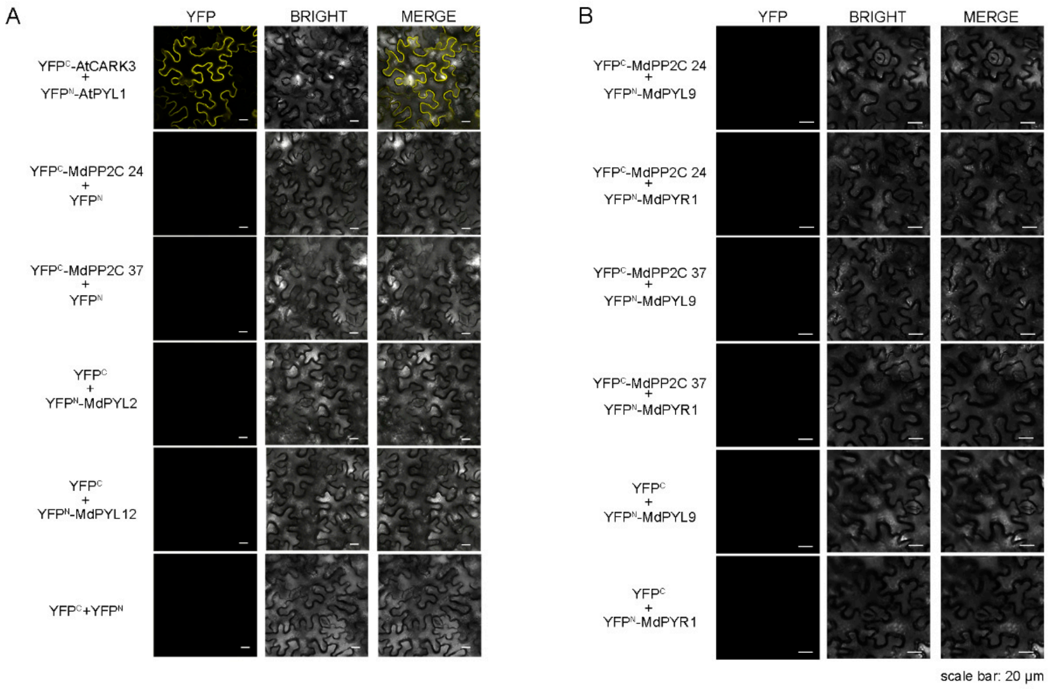

Figure S4. Positive control and negative results using bimolecular fluorescence complementation (BiFC) assay of this study. (A) The interaction of YFP^C–AtCARK3+YFP^N–AtPYL1 was used as positive control. The interaction of YFP^C–MdPP2C24/37+YFP^N and YFP^C+YFP^N–MdPYL2/12 and YFP^C+YFP^N were used as negative controls. (B) The interactions of YFP^C–MdPP2C24/37+YFP^N–MdPYL9/PYR1 were used as negative results. All experiments were replicated three times, with the same results obtained.

Table S1. List of Primers Used in This Study.

Primer's name	Primer sequences (5’→3’)	
RT-qPCR		
MdPP2C24 F	GCGGTTCAAATTCGTCTCCG	
MdPP2C24 R	TTTCCAACTCCCGAGAAGGC	
MdPP2C37 F	AGTTCTTGCCATGTCGAGGG	
MdPP2C37 R	GCAGGCGGTGTCGTTAGATA	
RAB18 F	CAGCAGCAGTATGACGAGTA	
RAB18 R	CAGTTCCAAAGCCTTCAGTC	
ERD10-F	TCTCTGAACCAGAGTCGTTT	
ERD10-R	CTTCTTCTCACCGTCTTCAC	
RD29A F	ATCACTTGGCTCCACTGTTGTTC	
RD29A R	ACAAAACACACATAAACATCCAAAGT	
KIN2 F	ACCAACAAGAATGCCTTCCA	
KIN2 R	ACTGCCGCATCCGATATACT	
ACTIN2 F	TTGACTACGAGCAGGAGATGG	
ACTIN2 R	ACAAACGAGGGCTGGAACAAG	
MdEF-1α-F	ATTCAAGTATGCCTGGGTGC	
MdEF-1α-R	CAGTCAGCCTGTGATGTTCC	
Plasmid Construct	Primer's name	Construct's name
MdPP2C24 F	GCTCTAGAATGGCGGAGACTTGCTACG	pBI221-*p35S:: MdPP2C24-GFP*<br>*Xba*I/*Sma*I
MdPP2C24 R	TCCCCCGGGCGCGTGTTGCGTCTGAGGT	
MdPP2C37 F	GCTCTAGAATGGCTGGAATGTGCTGCGG	pBI221-*p35S:: MdPP2C37-GFP*<br>*Xba*I/*Sma*I
MdPP2C37 R	TCCCCCGGGCGGGACCTCCCCGGTCTTCT	
MdPP2C24 F	GCTCTAGAATGGCGGAGACTTGCTACG	pCambia2300-*p35S:: MdPP2C24*<br>*XbaI/KpnI*
MdPP2C24 R	CCGGTACCCGTGTTGCGTCTGAGGT	
MdPP2C37 F	GCTCTAGAATGGCTGGAATGTGCTGCGG	pCambia2300-*p35S:: MdPP2C37*<br>*XbaI/KpnI*
MdPP2C37 R	CCGGTACCGGACCTCCCCGGTCTTCT	
MdPP2C24 F	GGAATTCCATATGATGGCGGAGACTTGCTACG	PGBKT7 (AD)-MdPP2C24<br>*NdeI/EcoRI*
MdPP2C24 R	CGGAATTCCTACGTGTTGCGTCTGAGGT	
MdPP2C37 F	GGAATTCCATATGATGGCTGGAATGTGCTGCGG	PGBKT7 (AD)-MdPP2C37<br>*NdeI/EcoRI*
MdPP2C37 R	CGGAATTCTTAGGACCTCCCCGGTCTTCT	
MdPYL2 F	CGGAATTCATGGATGCTAGCTCCGCTC	PGADT7 (BD)-MdPYL2<br>*EcoRI/PstI*
MdPYL2 R	AAAACTGCAGTGATTGATCATGTCCTCCCCC	
MdPYL9 F	TCCCCCCGGGATGAGCATCGGCGGCGG	PGADT7 (BD)-MdPYL9<br>*XmaI/PstI*
MdPYL9 R	AAAACTGCAGCTATTGATTGATAGGTTCGGTTCGG	
MdPYL12 F	GGAATTCCATATGATGTCGTACCCAACGTCCCA	PGADT7 (BD)-MdPYL12

MdPYL12 R	CGGAATTCTCAGCACGTAGCAGCCATC	*NdeI/EcoRI*
MdPYR F	TCCCCCCGGGATGGAGAAAAGCGAGGGC	PGADT7 (BD)-MdPYR1
MdPYR R	AAAACTGCAGTTAGCGCCCACCGTCAC	*XmaI/PstI*
MdPP2C24 F	GCTCTAGAATGGCGGAGACTTGCTACG	PSPYNE/PSPYCE-MdPP2C24
MdPP2C24 R	CCGGTACCCGTGTTGCGTCTGAGGT	*XbaI/KpnI*
MdPP2C37 F	GCTCTAGAATGGCTGGAATGTGCTGCGG	PSPYNE/PSPYCE-MdPP2C37
MdPP2C37 R	CCGGTACCGGACCTCCCCGGTCTTCT	*XbaI/KpnI*
MdPYL2 F	GCTCTAGAATGGATGCTAGCTCCGCTC	PSPYNE/PSPYCE-MdPYL2
MdPYL2 R	GGGGTACCTGATTGATCATGTCCTCCCCC	*XbaI/KpnI*
MdPYL12 F	GCTCTAGAATGTCGTACCCAACGTCCCA	PSPYNE/PSPYCE-MdPYL12
MdPYL12 R	GGGGTACCGCACGTAGCAGCCATC	*XbaI/KpnI*
